# Multi-Objective Grey Wolf Optimizer-Tuned LQR Attitude Control of a Three-DOF Hover System

**DOI:** 10.3390/biomimetics11030215

**Published:** 2026-03-17

**Authors:** Abdullah Çakan

**Affiliations:** Department of Mechanical Engineering, Konya Technical University, Selçuklu, Konya 42250, Türkiye; acakan@ktun.edu.tr

**Keywords:** multi-objective optimization, grey wolf optimizer (GWO), linear quadratic regulator (LQR), 3-DOF hover system, attitude control

## Abstract

Attitude control of unmanned aerial vehicles is a problem that needs to be solved in a reliable manner. The research presented in this paper examines a systematic approach to the design of an LQR state feedback controller for the three-DOF hover system. The state space model is used to derive the feedback gain K, with the diagonal elements of the weighting matrices Q and R used as design variables. A multi-objective grey wolf optimizer is used to obtain Q–R matrices based on closed-loop simulations under representative roll, pitch and yaw reference commands. There are four separate multi-objective optimization runs, each using one of four standard error indices which are the integral of absolute error (IAE), the integral of time-weighted absolute error (ITAE), the integral of squared error (ISE) and the integral of time-weighted squared error (ITSE). Each index is used to track roll, pitch and yaw errors at the same time and the resulting non-dominated solution sets are post-processed using TOPSIS to select a compromise knee-point design. The simulation results show that the adjusted LQR parameters lead to feasible tracking performance. The proposed framework provides a systematic and replicable method for LQR weight selection in hover-type attitude control problems under the considered simulation settings.

## 1. Introduction

The development of unmanned aerial vehicles (UAVs), specifically quadrotors, has experienced an exponential rise in the last decade owing to their remarkable versatility in a wide range of applications such as surveillance, logistics, search and rescue operations and environmental and scientific studies [1,2,3]. This rapid rise can be attributed to the advantages that quadrotors offer: vertical takeoff and landing, hovering stability, simplicity of design and maneuverability for fast direction changes and accurate positioning. The control of quadrotor systems is a fundamentally difficult task that is characterized by its natural underactuation (four control inputs for six degrees of freedom), strong coupling effects between the rotational axes, strong non-linearities in aerodynamics, fast response demands and operational constraints such as motor saturation and energy constraints [4].

The linear quadratic regulator (LQR) is the traditional optimal control solution for stabilizing complex linear systems like quadrotor attitude dynamics, ensuring guaranteed closed-loop stability, robustness margins and efficient real-time implementations for embedded flight control systems [5]. Nevertheless, the effectiveness of LQR control is highly sensitive to the choice of weighting matrices *Q* and *R*, which determine the essential trade-off between trajectory tracking accuracy and energy efficiency. The traditional manual tuning process, which relies on trial-and-error experimentation, lacks systematic methods and often results in suboptimal solutions, especially when multiple conflicting objectives need to be satisfied simultaneously [6]. A similar multi-criteria perspective has also been adopted in UAV controller design, where Pareto-based optimization has been used to balance multiple step-response objectives simultaneously in quadrotor tuning [7]. This inherent shortcoming has triggered extensive research efforts in the application of meta-heuristic optimization techniques for automating and improving the parameter selection process, which marked a paradigm shift in controller design methods.

Recent studies have clearly shown the effectiveness of bio-inspired algorithms in optimizing LQR parameters by searching high-dimensional parameter spaces. Comparative studies have also shown that several population-based optimization methods, including GA, DE, PSO and GWO, can be effectively used to determine the LQR weighting matrices of quadrotor trajectory-tracking controllers in a systematic manner [8]. Particle swarm optimization (PSO), inspired by the collective behavior of bird flocks, has been shown to be very effective in optimizing LQR weighting matrices using population-based search methods that do not require gradient computation [5]. In addition, genetic-algorithm-based optimization has also been employed for LQR weighting-matrix selection in quadrotor control problems, further demonstrating the usefulness of meta-heuristic search in reducing the reliance on manual tuning [9]. Comparative analyses of PSO, the flower pollination algorithm (FPA) and ant colony optimization (ACO) on the three-DOF hover platform show that FPA-based optimization outperforms others in terms of response speed with the least overshoot and hence is most useful in situations that demand fast transient response [10]. Grey wolf optimization (GWO) has recently emerged as a successful meta-heuristic, inspired by the complex social behavior and hunting strategies of grey wolf packs, which organizes the search population into a wolf pack hierarchy where alpha, beta and delta wolves correspond to solutions of increasing quality [11].

Comparative studies directly comparing PSO and GWO on the three-DOF hover system show clear differences in performance trade-offs: GWO-optimized LQR controllers show better disturbance rejection performance with less oscillation during external disturbances, while PSO-optimized controllers show better recovery speed with smoother voltage command trajectories. A hybrid algorithm incorporating simulated annealing (SA) with grey wolf optimization (hSA-GWO) has been proposed and tested on three-DOF systems, capitalizing on the fast convergence properties of SA and the excellent exploration abilities of GWO [12]. More recent studies have further shown that hybrid meta-heuristic structures can improve convergence quality and robustness in LQR-based quadrotor control by combining the exploration and exploitation capabilities of different search mechanisms [13]. Recent studies have also focused on improving the internal search mechanisms of MOGWO itself through enhanced population initialization, non-linear convergence control and archive update strategies in order to achieve better convergence accuracy and solution diversity in multi-objective engineering optimization problems [14]. The modified monarch butterfly optimization (M2BO) algorithm with distribution functions has been specifically proposed and experimentally tested on three-DOF hover systems, showing better control performance than traditional MBO algorithms and previous stochastic optimization methods like SMDO and DSO [15].

Sliding mode control (SMC) has been identified as a highly preferred method owing to its established robustness properties against parameter uncertainties and unmodeled dynamics, with robust implementations using combinatorial reaching laws that guarantee finite-time convergence and effectively mitigate the chattering effect using high-slope saturation functions in place of traditional sign functions [16]. The amplified linear quadratic regulator (ALQR) method integrates linear control topology with non-linear sliding mode control dynamics, which has shown better robustness properties against noise and unmodeled dynamics when implemented on the three-DOF system [17]. Simulink response optimization has been employed systematically to identify LQR weight matrices that minimize rise time, settling time and overshoot simultaneously, which enables automatic parameter tuning in conventional MATLAB settings [18]. In the case of systems with state delays and actuator faults, enhanced guaranteed cost control with quantum adaptive control ensures robust stability properties while preserving tracking performance.

Hamilton–Jacobi–Bellman (HJB) equation-based optimal control represents a sophisticated approach to multi-rotor attitude control that explicitly handles system non-linearities, with multiple design methods based on the HJB equation including linear control with stability guarantees for non-linear systems and non-linear suboptimal control techniques that have been developed and experimentally evaluated on the three-DOF Hover [19]. The experimental evaluation reveals important practical insights: linear control methods often outperform theoretically more sophisticated non-linear strategies in real-world implementation, suggesting that the practical trade-offs between theoretical optimality and implementation complexity must be carefully considered in control system design. Artificial neural networks (ANNs) have been employed to approximate complex system non-linearities, simplifying the design of both LQR and SMC controllers for highly non-linear systems [20]. Sophisticated non-linear control approaches for twin rotor MIMO systems (TRMSs) representing two-DOF helicopter configurations have been developed, including dual boundary conditional integral backstepping control (DBCIBC) that provides an innovative modification of integral backstepping techniques to ensure efficient asymptotic output regulation without degrading transient response [21]. This approach decouples the helicopter-like system into vertical and horizontal subsystems with cross couplings considered as uncertainties, enabling robust output regulation in the presence of system uncertainties and external disturbances.

Stochastic optimization algorithms have been employed to fine-tune feedback gains for multi-rotor systems in stochastic environments. Ates et al. employed stochastic multi-parameter divergence optimization (SMDO) and discrete stochastic optimization (DSO) to improve the stability of micro-platforms and showed the effectiveness of their approach over traditional LQR methods using probabilistic convergence and robustness guarantees, which are highly beneficial for systems that must function in uncertain environments. Fuzzy logic controllers with meta-heuristic optimization of parameters have been shown to be effective in model uncertainty while ensuring stability margins [22].

Besides control design, a real-world quadrotor system must also address fault detection, diagnosis and fault tolerance. Methods of fault diagnosis based on generalized Hamiltonian system models have been proposed for robotic systems and directly applied to two-DOF helicopter robots for the effective detection and isolation of particular failure modes by decomposing the original system into subsystems that are sensitive to particular faults [23]. Passive fault-tolerant controllers based on robust control methods have been proposed and tested on two-DOF robotic helicopters by using linear matrix inequalities to modify proportional-derivative control methods and adding non-linear terms to enhance the overall control accuracy [24].

Recent developments in machine learning and adaptive control have brought about new ideas for designing quadrotor controllers. Learning-based robust trajectory tracking control of two-DOF helicopter systems using gradient descent-based learning control laws aims to minimize cost functions associated with desired closed-loop error dynamics of non-linear systems, where the learning ability of the controller makes it more suitable for dealing with system uncertainties and unknown disturbances during online flight operations [25]. Reinforcement learning (RL)-based approaches, such as deep deterministic policy gradient (DDPG) algorithms, have been successfully utilized for online fine-tuning of proportional-derivative controller parameters, thereby filling the large gap between simulation-trained controllers and real-world quadrotor control applications [26].

State estimation plays an ever more important role with increasing system complexity, as in real-world applications, not all states of the system can be measured directly by the sensors on board, which has a profound effect on control system performance and closed-loop stability. Observer designs of both full and reduced order have been successfully employed on two-DOF helicopter models to stabilize the system and achieve real-time state estimation [27]. For two-DOF systems with strong non-linearities, neural network-based observers have been designed that do not need any prior knowledge of the system dynamics, employing two-layer neural networks to model the strong non-linearities of complex systems and state estimation methods become even more important for three-DOF systems, where the higher dimensionality makes it increasingly impractical to measure all states directly [28].

The three-DOF hover platform is not only used for research purposes but is also a valuable educational platform for teaching advanced control concepts to students and researchers. Educational platforms for non-linear control systems focus on the improvement of student motivation and learning through specific curricula that include comprehensive experimental approaches and the utilization of advanced platforms such as the Quanser hover allows students and researchers to gain conceptual knowledge while also providing hands-on experience with advanced control implementations [29]. Laboratory manuals for MATLAB/Simulink users include detailed information on how to derive state-space models of open-loop systems, as well as the design and implementation of LQR-based state-feedback controllers and the simulation of systems to ensure stabilization within realistic constraints [30].

Although several meta-heuristic approaches have previously been reported for tuning LQR weights on these kind of systems, the purpose of this study is not to claim a universally superior controller. Instead, the novelty of this work lies in examining the applicability of a multi-objective grey wolf variant for systematic LQR weight selection under practical implementation constraints. In particular, a multi-objective grey wolf optimizer (MOGWO) is used to treat roll, pitch and yaw tracking errors simultaneously as a three-objective problem. Moreover, the tuning and evaluation are carried out within the same closed-loop MATLAB/Simulink environment so that the reported designs reflect feasible implementation conditions.

Accordingly, the contributions of this paper are as follows: (i) a reproducible MOGWO-LQR tuning workflow for the coupled three-DOF hover attitude model; (ii) four independent multi-objective runs based on standard error indices, each defined concurrently for roll, pitch and yaw; and (iii) a Pareto-set and knee-point-based selection of a single implementable (Q,R) design that is subsequently assessed through step and scenario tracking simulations.

**Figure 1:**
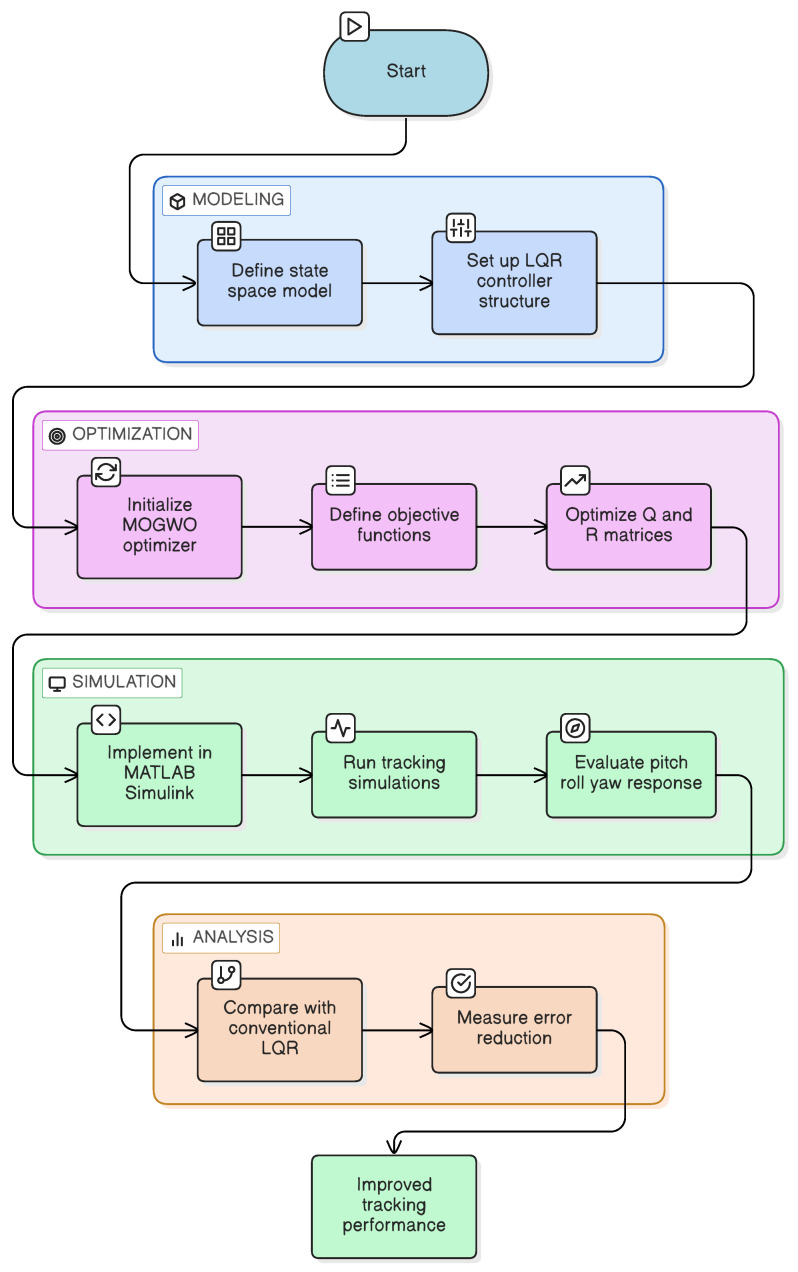
Flow chart of the proposed methodology.

The overall workflow of the proposed methodology is summarized in the flowchart presented in Figure 1.

## 2. Three-DOF Hover System Description and Modeling

**Figure 2:**
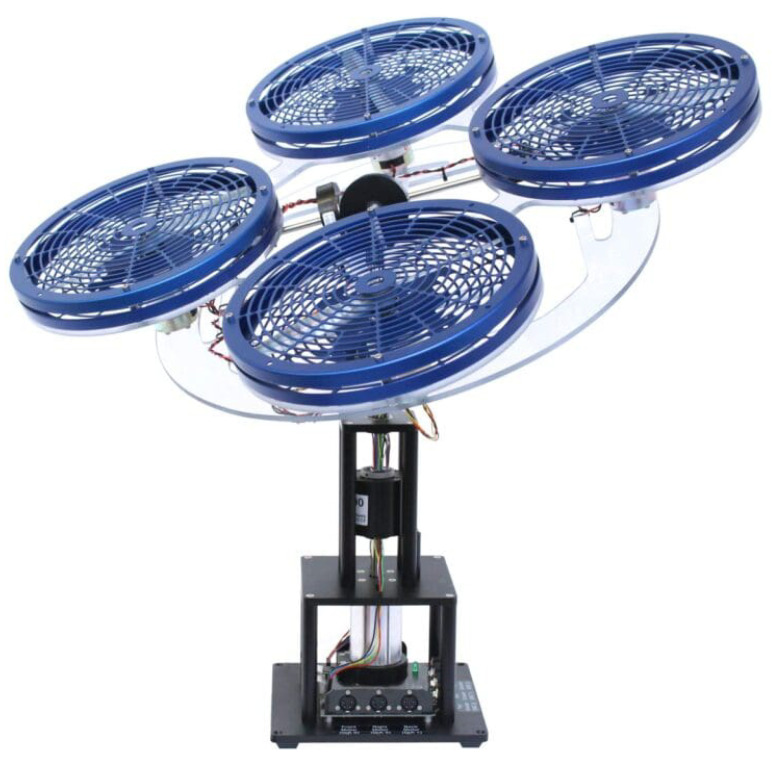
3 DOF hover system [34].

The three-DOF hover system has emerged as the standard experimental testbed for validating flight control algorithms in a safe laboratory environment, enabling systematic investigation of control performance under realistic constraints, as shown in Figure 2. This electromechanical system accurately models the rotational dynamics characteristic of quadrotor and tandem helicopter aircraft by controlling three critical angular degrees of freedom which are the pitch ($\theta_{p}$), roll ($\theta_{r}$) and yaw ($\theta_{y}$) angles through independent motor-driven propeller mechanisms [30,31]. The three-DOF platform represents a significant advancement over earlier two-DOF helicopter systems by enabling comprehensive investigation of three-axis attitude dynamics with their complex interdependencies [32,33].

**Figure 3:**
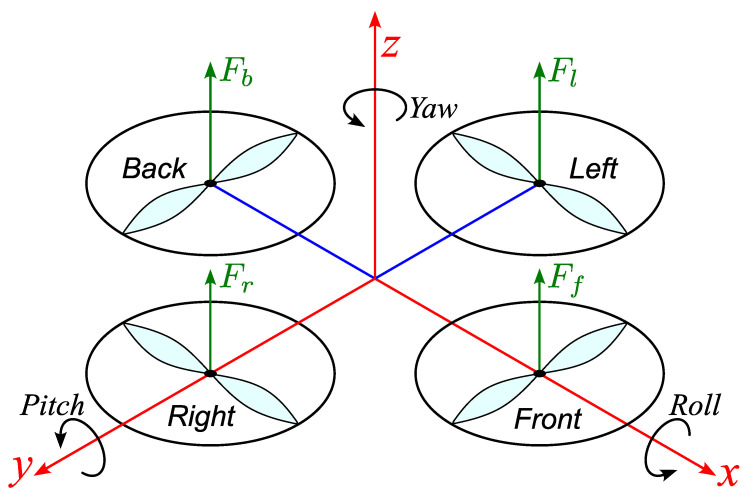
Schematic diagram of three-DOF hover system.

The schematic diagram of the three-DOF hover system is shown in Figure 3. Throughout this study, the following modeling conventions are adopted to ensure consistent sign definitions: the system is horizontal when $\theta_{p}=0$ and $\theta_{r}=0$. Yaw angle increases, $\theta_{y}>0$, when the body rotates in the CCW direction. Pitch angle increases, $\theta_{p}>0$, when the body rotates the CCW direction. Roll angle increases, $\theta_{r}>0$, when the body rotates the CCW direction.

When a positive voltage is applied to any motor, a positive thrust force is generated and the corresponding propeller assembly rises. The thrust forces produced by the front, back, right and left motors are denoted by $F_{f}$, $F_{b}$, $F_{r}$ and $F_{l}$, respectively and the applied motor voltages are $V_{f}$, $V_{b}$, $V_{r}$ and $V_{l}$. Pitch motion is primarily governed by the differential thrust between the front and back motors, while roll motion is driven mainly by the differential thrust between the right and left motors. Accordingly, the pitch angle increases when $F_{f}>F_{b}$ and the roll angle increases when $F_{r}>F_{l}$.

The rotational dynamics around each axis are expressed using the general form given in Equation (1).

(1) $$J\ddot{\theta }=\Delta F\;L,$$

where θ is the angular displacement, *L* is the distance from the pivot to each motor along the corresponding axis, *J* is the equivalent moment of inertia about the axis and ΔF denotes the differential thrust force.

**Figure 4:**
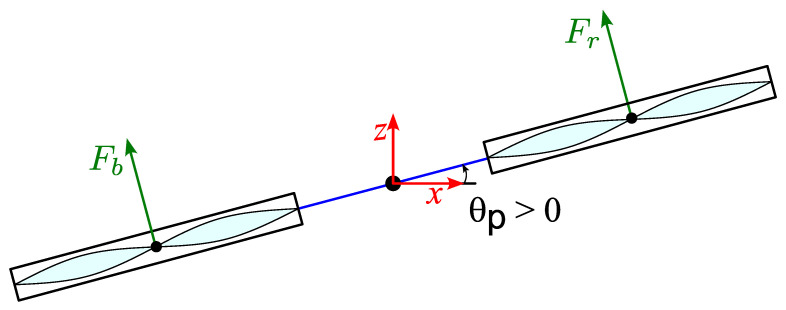
Schematic diagram of pitch axis.

Using the schematic diagram of the pitch axis in Figure 4, the pitch and similarly roll equations of motion are given in Equations (2) and (3), respectively.

(2) $$J_{p}{\ddot{\theta }}_{p}=K_{f}\left(V_{f}-V_{b}\right),$$

(3) $$J_{r}{\ddot{\theta }}_{r}=K_{f}\left(V_{l}-V_{r}\right),$$

where $K_{f}$ is the thrust force constant, $V_{f}$ is the front motor voltage, $V_{b}$ is the back motor voltage, $V_{r}$ is the right motor voltage, $V_{l}$ is the left motor voltage, $\theta_{p}$ is the pitch angle, $\theta_{r}$ is the roll angle, $J_{p}$ is the moment of inertia about the pitch axis and $J_{r}$ is the moment of inertia about the roll axis.

**Figure 5:**
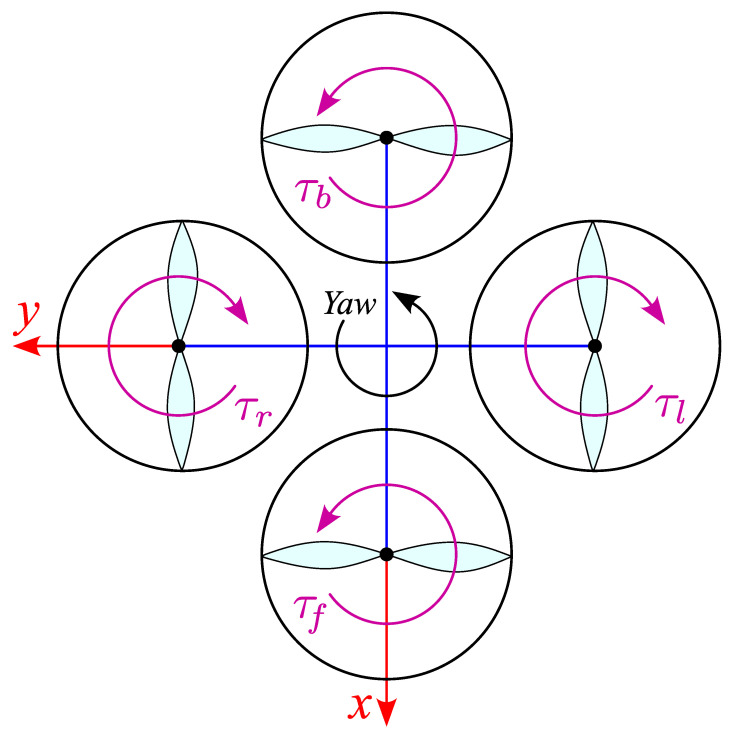
Schematic diagram of yaw axis.

The yaw motion is caused by the imbalance of reaction torques in Equation (4) generated by the two clockwise and two counter-clockwise rotors shown in Figure 5. Assuming a linear mapping between motor voltage and propeller torque, $\tau =K_{t}V_{m}$, the yaw axis equation of motion can be written in terms of applied motor voltages as in Equation (5).

(4) $$J_{y}{\ddot{\theta }}_{y}=\Delta \tau =\tau_{l}+\tau_{r}-\tau_{f}-\tau_{b},$$

(5) $$J_{y}{\ddot{\theta }}_{y}=K_{t}\left(V_{r}+V_{l}\right)-K_{t}\left(V_{f}+V_{b}\right).$$

The resulting linear model is formulated in the standard state-space form

(6) $$\dot{x}=Ax+Bu,$$

(7) y=Cx+Du.

where the state, input and output vectors are defined in Equations (8) and (10); the corresponding matrices (A,B,C,D) are given in Equations (11) and (12).

(8) $$x^{⊤}=\left[\begin{array}{cccccc} \theta_{y} & \theta_{p} & \theta_{r} & {\dot{\theta }}_{y} & {\dot{\theta }}_{p} & {\dot{\theta }}_{r} \end{array}\right],$$

(9) $$u^{⊤}=\left[\begin{array}{cccc} V_{f} & V_{b} & V_{r} & V_{l} \end{array}\right].$$

(10) $$y^{⊤}=\left[\begin{array}{ccc} \theta_{y} & \theta_{p} & \theta_{r} \end{array}\right],$$

(11) $$A=\left[\begin{array}{cccccc} 0 & 0 & 0 & 1 & 0 & 0\\ 0 & 0 & 0 & 0 & 1 & 0\\ 0 & 0 & 0 & 0 & 0 & 1\\ 0 & 0 & 0 & 0 & 0 & 0\\ 0 & 0 & 0 & 0 & 0 & 0\\ 0 & 0 & 0 & 0 & 0 & 0 \end{array}\right],\;B=\left[\begin{array}{cccc} 0 & 0 & 0 & 0\\ 0 & 0 & 0 & 0\\ 0 & 0 & 0 & 0\\ -\frac{K_{t}}{J_{y}} & -\frac{K_{t}}{J_{y}} & \frac{K_{t}}{J_{y}} & \frac{K_{t}}{J_{y}}\\ \frac{LK_{f}}{J_{p}} & -\frac{LK_{f}}{J_{p}} & 0 & 0\\ 0 & 0 & \frac{LK_{f}}{J_{r}} & -\frac{LK_{f}}{J_{r}} \end{array}\right],$$

(12) $$C=\left[\begin{array}{cccccc} 1 & 0 & 0 & 0 & 0 & 0\\ 0 & 1 & 0 & 0 & 0 & 0\\ 0 & 0 & 1 & 0 & 0 & 0 \end{array}\right],\;D=\left[\begin{array}{cccc} 0 & 0 & 0 & 0\\ 0 & 0 & 0 & 0\\ 0 & 0 & 0 & 0 \end{array}\right].$$

The mathematical model considered in this study, together with the associated physical parameters, is based on the Quanser three-DOF hover user documentation [30]. Therefore, the governing equations presented in this section are adopted from this reference and are provided to describe the system model used for controller design and simulation. The physical parameters employed in the model are summarized in Table 1.

## 3. LQR Controller Design

The design of a full-state feedback controller for regulating the attitude of the three-DOF hover system is presented in this section. Using the linear state-space model derived in the previous section, an LQR-based gain matrix is computed to track the desired yaw, pitch and roll angles while limiting the required actuator effort.

The motor command vector is chosen as the four motor voltages,

(13) $$u={\left[\begin{array}{cccc} V_{f} & V_{b} & V_{r} & V_{l} \end{array}\right]}^{\;⊤}=\left\{\begin{array}{cc} K\;\left(x_{d}-x\right)+u_{\mathrm{bias}}, & \mathrm{if}\;u\geq 0,\\ 0, & \mathrm{if}\;u<0, \end{array}\right.$$

where *x* is the state vector defined in (8) and $K\in R^{4\times 6}$ is the state-feedback gain. The reference (setpoint) vector is defined as

(14) $$x_{d}=\left[\begin{array}{cccccc} \theta_{d,y} & \theta_{d,p} & \theta_{d,r} & 0 & 0 & 0 \end{array}\right],$$

which specifies the desired yaw, pitch and roll angles with zero desired angular rates. A constant bias voltage is added to each motor,

(15) $$u_{\mathrm{bias}}^{⊤}=\left[\begin{array}{cccc} V_{\mathrm{bias}} & V_{\mathrm{bias}} & V_{\mathrm{bias}} & V_{\mathrm{bias}} \end{array}\right],$$

to keep the propellers spinning and to prevent command voltages from dropping below the cutoff region, thereby improving responsiveness around the hover operating point. In implementation, the non-negativity constraint in (13) is enforced elementwise and the actuator voltage is additionally limited to the available supply range in Simulink.

Due to the low resistance of the motors, frequent switching between positive and negative voltages may lead to excessive current and can damage the power amplifier. Therefore, only non-negative voltages are applied. This constraint is also consistent with practical VTOL/helicopter systems, where propellers do not reverse direction during normal operation.

The LQR gain is computed based on the standard feedback law [35]

(16) u=−Kx,

where the weighting matrices are parameterized as diagonal matrices:

(17) $$Q=\left[\begin{array}{cccccc} q_{11} & 0 & 0 & 0 & 0 & 0\\ 0 & q_{22} & 0 & 0 & 0 & 0\\ 0 & 0 & q_{33} & 0 & 0 & 0\\ 0 & 0 & 0 & q_{44} & 0 & 0\\ 0 & 0 & 0 & 0 & q_{55} & 0\\ 0 & 0 & 0 & 0 & 0 & q_{66} \end{array}\right],\;R=\left[\begin{array}{cccc} r_{11} & 0 & 0 & 0\\ 0 & r_{22} & 0 & 0\\ 0 & 0 & r_{33} & 0\\ 0 & 0 & 0 & r_{44} \end{array}\right].$$

Although the three-DOF hover dynamics are coupled, *Q* and *R* are parameterized as diagonal matrices in (17) for a practical and reproducible tuning setup. This choice reduces the number of decision variables and preserves interpretability by assigning independent penalties to each state and each motor-voltage input. It also simplifies enforcing the required properties Q≥0 and R>0 through non-negative diagonal entries, which is convenient for meta-heuristic search. Importantly, using diagonal weights does not imply a decoupled controller: since coupling is already embedded in the plant matrices (A,B), the resulting LQR gain *K* is generally dense and can still coordinate multiple states and inputs.

Allowing off-diagonal terms could further influence the obtained controller by explicitly penalizing state cross-products and input correlations. Nonzero $q_{ij}$ terms would weight coupled state combinations and may change how the controller trades off cross-axis interactions, while nonzero $r_{ij}$ terms could penalize correlated motor-command combinations. Such extensions would increase the search dimension and reduce interpretability and their impact would depend on the selected structure and bounds.

Here, *Q* penalizes the state deviations (attitude angles and rates), whereas *R* penalizes the control effort in terms of motor voltages. Using (A,B) from (11), the gain *K* is obtained by minimizing the quadratic performance index [35]

(18) $$J=\int_{0}^{\infty }\left(x^{⊤}Qx+u^{⊤}Ru\right)\;dt.$$

## 4. MOGWO-Based Optimization of LQR Parameters

**Figure 6:**
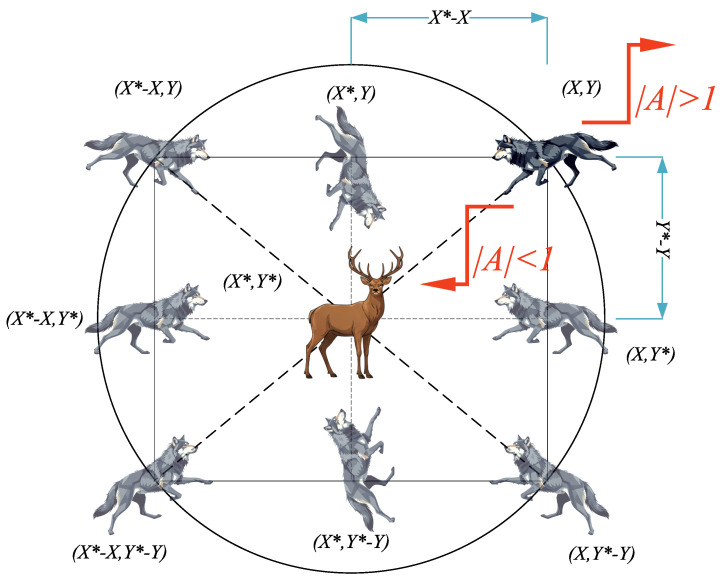
Position updating strategy. *X* and *Y* denote the current search-agent coordinates, $X^{*}$ and $Y^{*}$ represent the estimated target coordinates and |A| controls exploration and exploitation [37,38].

In this study, the multi-objective grey wolf optimizer (MOGWO) is used to optimize the diagonal entries of the LQR weighting matrices in (17), following a parameterization similar to that in [36]. In practice, the diagonal entries are tuned within the predefined bounds given in Table 2 and for each candidate set of (Q,R), the gain matrix *K* is computed via the LQR formulation in (18). The grey wolf optimizer (GWO) was introduced by Mirjalili et al. [37], inspired by the leadership hierarchy and hunting behavior of grey wolves, where the best three candidate solutions (α, β, δ) guide the remaining wolves (ω). MOGWO extends GWO to multi-objective problems by employing an external archive to store non-dominated solutions and an adaptive grid mechanism to preserve diversity in the objective space, as shown in Figure 6 [38].

Four separate multi-objective optimization studies were performed. In the first study, the objective vector was defined using ITAE for the roll, pitch and yaw channels:

(19) $$F_{\mathrm{ITAE}}=\left[{\mathrm{ITAE}}_{\mathrm{roll}},\;{\mathrm{ITAE}}_{\mathrm{pitch}},\;{\mathrm{ITAE}}_{\mathrm{yaw}}\right].$$

**Figure 7:**
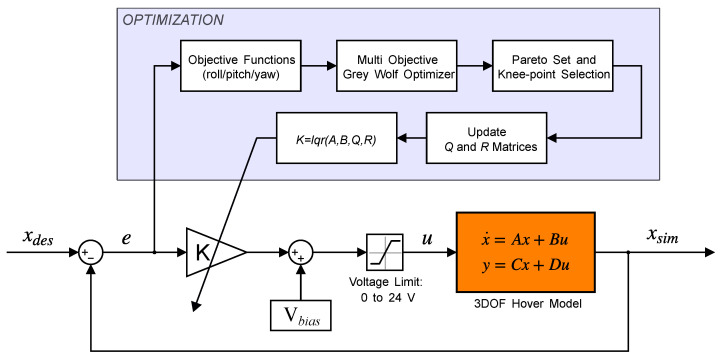
Block diagram of the MOGWO-based LQR tuning loop and the closed-loop three-DOF hover control implementation.

The same three-channel multi-objective formulation was then repeated in three additional independent optimization runs using IAE, ITSE and ISE, respectively. The performance indices for each attitude channel i∈{roll,pitch,yaw} are defined as

(20) $${\mathrm{ITAE}}_{i}=\int_{0}^{T}t\;\left|e_{i}\left(t\right)\right|\;dt,$$

(21) $${\mathrm{IAE}}_{i}=\int_{0}^{T}\left|e_{i}\left(t\right)\right|\;dt,$$

(22) $${\mathrm{ITSE}}_{i}=\int_{0}^{T}t\;e_{i}^{2}\left(t\right)\;dt,$$

(23) $${\mathrm{ISE}}_{i}=\int_{0}^{T}e_{i}^{2}\left(t\right)\;dt,$$

where $e_{i}\left(t\right)$ denotes the tracking error of the *i*-th attitude channel and *T* is the simulation horizon. Each run yields a set of *Q*, *R*; a single implementable solution can then be selected from the Pareto set. The overall optimization control workflow is illustrated in Figure 7.

The encircling behavior is modeled as

(24) $$\vec{D}=\left|\vec{C}\cdot {\vec{X}}_{p}\left(t\right)-\vec{X}\left(t\right)\right|$$

(25) $$\vec{X}\left(t+1\right)={\vec{X}}_{p}\left(t\right)-\vec{A}\cdot \vec{D},$$

where *t* is the iteration index, $\vec{X}$ is the position of a search agent and ${\vec{X}}_{p}$ represents the prey (leader) position. The coefficient vectors are

(26) $$\vec{A}=2\vec{a}\cdot {\vec{r}}_{1}-\vec{a},$$

(27) $$\vec{C}=2\cdot {\vec{r}}_{2},$$

with $\vec{a}$ decreasing linearly from 2 to 0 and ${\vec{r}}_{1},{\vec{r}}_{2}\in \left[0,1\right]$.

The hunting mechanism updates each agent with respect to the three leaders α, β and δ, as shown in Equations (28)–(34):

(28) $${\vec{D}}_{\alpha }=\left|{\vec{C}}_{1}\cdot {\vec{X}}_{\alpha }-\vec{X}\right|,$$

(29) $${\vec{D}}_{\beta }=\left|{\vec{C}}_{2}\cdot {\vec{X}}_{\beta }-\vec{X}\right|,$$

(30) $${\vec{D}}_{\delta }=\left|{\vec{C}}_{3}\cdot {\vec{X}}_{\delta }-\vec{X}\right|,$$

(31) $${\vec{X}}_{1}={\vec{X}}_{\alpha }-{\vec{A}}_{1}\cdot \left({\vec{D}}_{\alpha }\right),$$

(32) $${\vec{X}}_{2}={\vec{X}}_{\beta }-{\vec{A}}_{2}\cdot \left({\vec{D}}_{\beta }\right),$$

(33) $${\vec{X}}_{3}={\vec{X}}_{\delta }-{\vec{A}}_{3}\cdot \left({\vec{D}}_{\delta }\right),$$

(34) $$\vec{X}\left(t+1\right)=\frac{{\vec{X}}_{1}+{\vec{X}}_{2}+{\vec{X}}_{3}}{3}.$$

As highlighted in Figure 6 of the original MOGWO study, the magnitude of $\vec{A}$ controls the exploration–exploitation behavior: when $|\vec{A}|>1$, the agents tend to explore (diverge) and when $|\vec{A}|<1$, they tend to exploit (converge).

Table 2 reports the baseline values adopted from the system documentation [30] together with the minimum and maximum bounds used for the decision variables in the optimization, namely the diagonal entries of the LQR weighting matrices *Q* and *R*.

- Randomly generate a set of candidate solutions (wolves), each encoding the selected entries of (Q,R) within the bounds given in Table 2.
- For every candidate, construct *Q* and *R*, compute *K* via LQR, simulate the closed loop and compute the three objective values.
- Identify non-dominated solutions and store them in an external archive. If a new candidate is dominated by an archive member, it is discarded; if it dominates archive members, those are removed; otherwise, it is added to the archive.
- When the archive reaches its maximum capacity, apply the grid and segmentation mechanism in the objective space and remove a solution from the most crowded region, then keep/insert solutions to favor less crowded regions.
- Choose α, β and δ from the archive using a diversity-aware leader-selection strategy biased toward less crowded regions.
- Update each wolf position using Equations (28)–(34), then update $\vec{a}$, $\vec{A}$ and $\vec{C}$.
- Repeat objective evaluation, archive update, diversity control, leader selection and position update until the stopping criterion is met; finally, return the archive as the approximated Pareto set.

To select a single implementable design from the final non-dominated Pareto archive, this study employs the Technique for Order Preference by Similarity to Ideal Solution (TOPSIS) [39,40]. In each optimization run, the external archive is limited to $N_{A}=100$ non-dominated solutions and TOPSIS is applied to rank these candidates based on their relative closeness to an ideal best and an ideal worst solution defined in the objective space. The highest-ranked candidate is taken as the compromise solution and used in subsequent simulations.

In this study, all objectives are minimization-type and correspond to the roll, pitch and yaw tracking indices for the selected performance measure. The same TOPSIS-based selection procedure was applied consistently for all four optimization runs, yielding one representative (Q,R) design from the final Pareto archive for each case.

All simulations were performed in a MATLAB/Simulink closed-loop hover model using the ode1 (Euler) solver with a fixed step size of 0.001 s. For the unit-step response analyses, the simulation horizon was set to 4 s and a unit step of amplitude 1 was applied separately to each attitude channel, while the remaining references were kept at zero. For the multi-axis validation scenario, the simulation horizon was 20 s, with reference commands of 4° for roll, 4° for pitch and 5° for yaw. All states were initialized to zero. Each motor voltage was independently constrained within the range of 0–24 V, with negative control commands clipped to 0 V and values above 24 V clipped to 24 V. No explicit fixed random seed was imposed and the stochastic search process followed MATLAB’s current random-number-generator state. To facilitate reproducibility, the MOGWO implementation followed the standard archive-grid mechanism and leader selection strategy described in the original MOGWO study [38]. Table 3 lists the optimizer parameters used to evaluate each candidate (Q,R) through closed-loop MATLAB/Simulink runs.

Following the above procedure, four independent MOGWO runs are completed for the ITAE-, IAE-, ITSE- and ISE-based objective formulations. The resulting optimized diagonal entries of the LQR weighting matrices *Q* and *R* are reported in Table 4. These values are subsequently used in the closed-loop simulations.

## 5. Results and Discussion

The results presented in this section summarize the simulation outcomes of the proposed MOGWO-LQR tuning framework and discuss its applicability to LQR weight selection for a coupled three-axis hover-type attitude system under practical actuator constraints. The discussion is structured to first present the step-response behavior of the optimized controllers then report the corresponding error index values and finally compare the obtained responses with baseline settings and representative meta-heuristic approaches reported in the literature.

**Figure 8:**
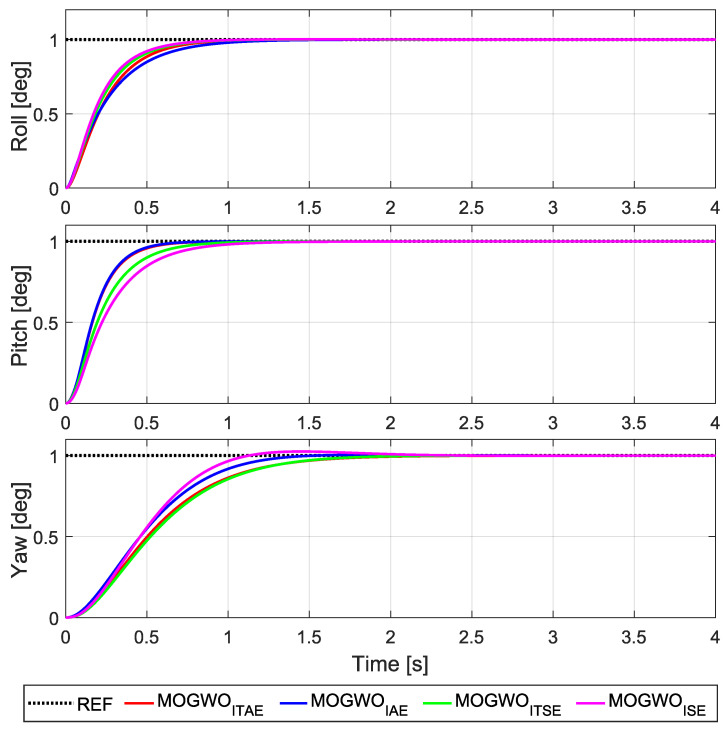
Step responses of roll, pitch and yaw for the MOGWO-tuned LQR designs.

Figure 8 presents the step responses obtained with the LQR gains computed from the MOGWO-optimized (Q,R) sets, where the roll, pitch and yaw channels are evaluated under the same voltage saturation and non-negativity constraints used during tuning. These plots provide a direct view of the transient behavior produced by the selected compromise solutions for each objective formulation.

Overall, the optimized designs yield stable tracking responses with channel-dependent trade-offs. While some solutions emphasize faster convergence in a given axis, others exhibit smoother transients with reduced oscillation. This behavior is expected because the knee-point selection balances three coupled attitude channels rather than prioritizing a single axis.

To quantify the trends observed in the step responses, the corresponding error-based performance indices computed over the simulation horizon are summarized in Table 5. The reported values represent the objective function outcomes associated with the step responses in Figure 8.

The results confirm that changing the objective formulation leads to different compromise solutions across roll, pitch and yaw. Improvements in one channel or index do not necessarily translate into uniform reductions in the remaining indices, which is consistent with the coupled nature of the plant and the multi-objective selection of (Q,R).

**Figure 9:**
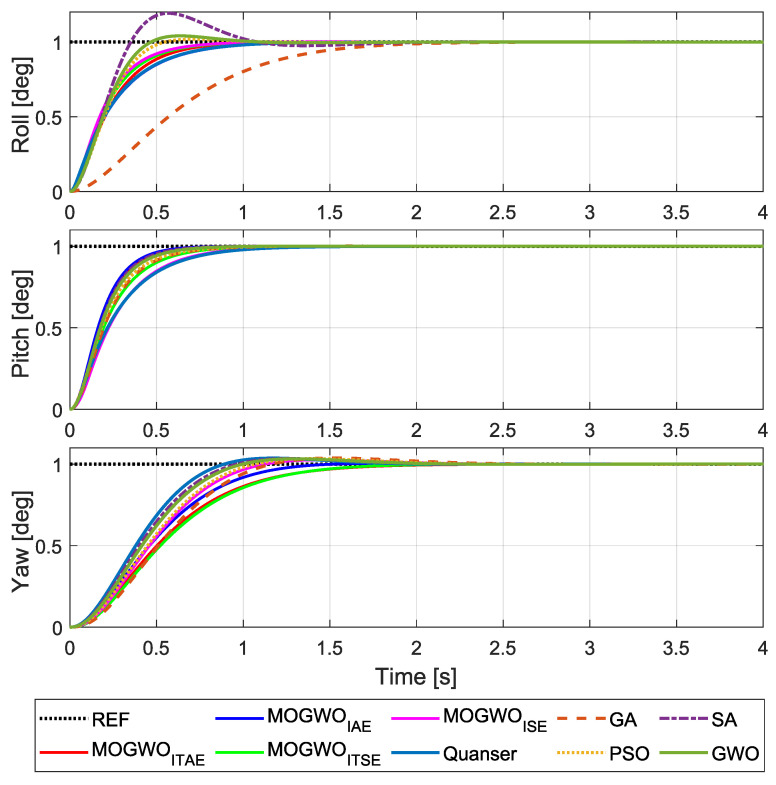
Step-response comparison with documentation baseline and literature methods [12,30].

For a broader perspective, Figure 9 compares the step responses of the proposed MOGWO-tuned LQR designs, with representative results reported in the literature using GA, PSO, SA and GWO, as well as the baseline LQR settings provided in the system documentation. This comparison is intended to contextualize the obtained responses rather than to claim a universally superior method, since the compared studies may differ in tuning settings and evaluation conditions.

The comparison indicates that the MOGWO-based tuning can produce step responses that are competitive in terms of transient behavior across the three axes. At the same time, differences among methods highlight that the final response characteristics depend strongly on how the objective functions, constraints and selection criteria are defined.

To complement the visual comparison in Figure 9, Table 6 reports standard step-response metrics for the considered controllers. The table enables a more direct assessment of transient characteristics such as settling time and overshoot across roll, pitch and yaw.

The numerical metrics support the qualitative observations from Figure 9. In general, the proposed tuning framework can reduce selected transient measures in some axes while maintaining feasible responses.

**Figure 10:**
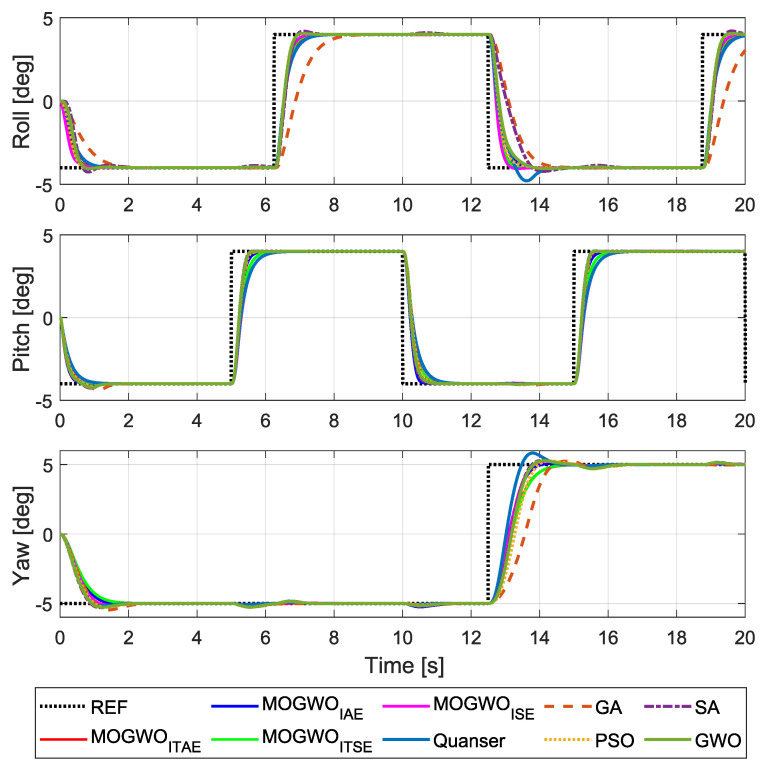
Reference tracking scenario showing cross-axis coupling effects.

Finally, Figure 10 illustrates the system response under a representative multi-axis scenario adopted from the system documentation, comparing the proposed approach with the baseline and literature-based controllers [12,30].

As observed in Figure 10, axis coupling leads to cross-axis interactions that cannot be fully eliminated by independent tuning of single-axis behavior. The proposed MOGWO-based tuning provides a practical way to search for (Q,R) choices that maintain acceptable multi-axis behavior in the presence of these interactions. This figure is particularly useful for highlighting coupling effects: when one axis is commanded, the induced motion may act as a disturbance on the other axes.

**Figure 11:**
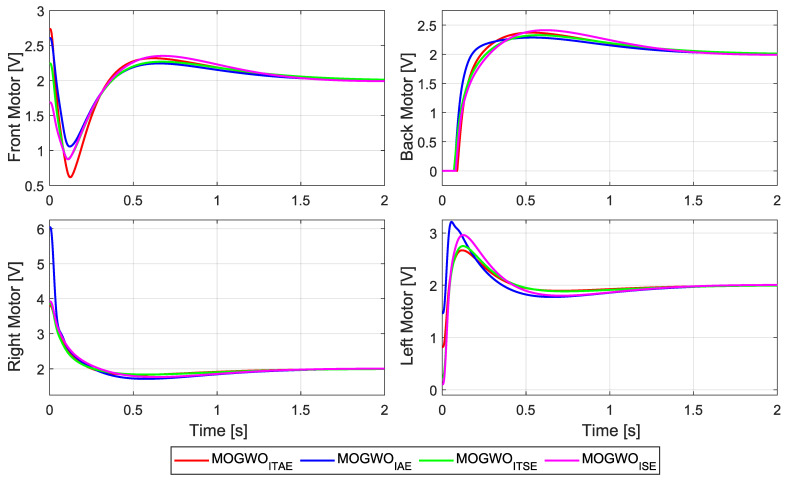
Motor-voltage commands during step-response tests for the MOGWO-tuned LQR designs.

The motor-voltage profiles corresponding to the step-response evaluations are shown in Figure 11. The four objective formulations lead to slightly different transient voltage demands, especially during the initial response where the controller reacts to the reference change. Despite these differences, all cases exhibit similar steady-state voltage levels and remain within the enforced actuator constraints, indicating that the selected MOGWO-based designs achieve the desired tracking behavior with comparable voltage usage across the front, back, right and left motors. A quantitative comparison of the motor-voltage signals is also provided in Table 7, where the minimum, peak, mean and RMS voltage values confirm that the overall actuator effort remains at a comparable level for all four controllers.

**Figure 12:**
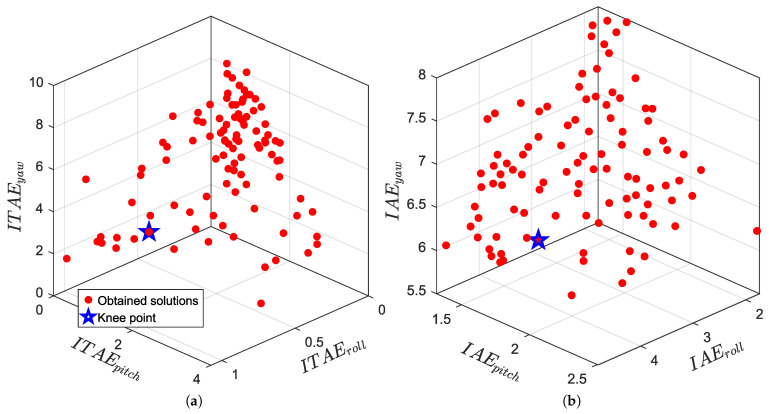

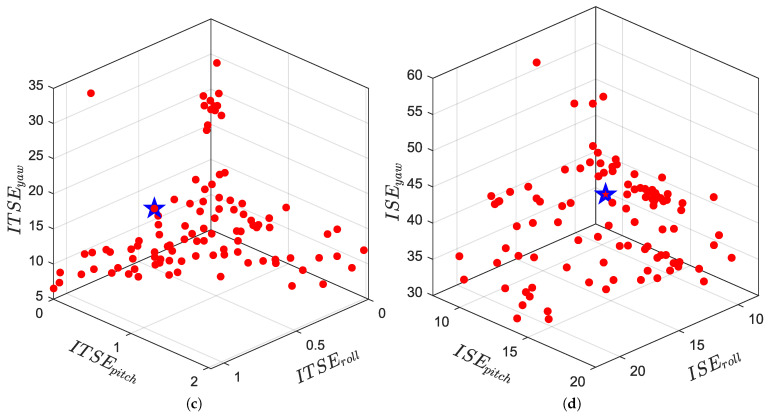
Pareto fronts and selected knee-point for the four runs: (**a**) ITAE, (**b**) IAE, (**c**) ITSE, (**d**) ISE.

In addition to time-domain responses, it is useful to visualize how the multi-objective search distributes candidate solutions in the objective space. Figure 12 shows the three-dimensional Pareto distributions obtained from the four independent optimization runs, where each run considers the roll, pitch and yaw indices simultaneously under the same simulation settings and actuator constraints. The red points denote the non-dominated solutions retained in the archive and the blue marker indicates the selected compromise (knee-point) solution used for subsequent simulations.

As seen in Figure 12, each objective formulation leads to a distinct spread of non-dominated solutions, reflecting different trade-offs among roll, pitch and yaw tracking errors. The presence of a well-populated set of alternatives suggests that the optimizer can produce multiple feasible (Q,R) candidates rather than converging to a single narrow region. The knee-point selection provides a practical way to choose one implementable design from the archive by balancing the three axes, which is particularly relevant for this coupled system where improving one channel may influence the others.

## 6. Conclusions

This study investigates the applicability of a multi-objective meta-heuristic approach for systematic tuning of LQR weighting matrices in a coupled three-DOF hover attitude control problem. The diagonal entries of the *Q* and *R* matrices are treated as decision variables within predefined bounds and a multi-objective grey wolf optimizer is integrated with closed-loop MATLAB/Simulink simulations to generate feasible candidate solutions. Rather than relying on manual trial-and-error, the proposed workflow produced non-dominated Pareto sets and enabled the selection of a single implementable design via a compromise knee-point selection.

The results show that the multi-objective formulation provides a structured way to balance roll, pitch and yaw tracking behavior simultaneously, which is particularly relevant for this system due to axis coupling. Across the independent runs based on standard error indices, the optimizer yielded different trade-off distributions in the objective space and corresponding LQR weight patterns, indicating that the selected objective definition can influence the resulting compromise design. When compared with baseline settings and representative single objective meta-heuristic designs reported in the literature, the MOGWO-tuned solutions exhibited competitive step and scenario-tracking behavior while remaining consistent with the implemented non-negativity and saturation constraints. These outcomes suggest that multi-objective grey wolf optimization can be used as a practical tuning tool for LQR weights in hover-type attitude systems, without claiming universal superiority over alternative methods.

Future work will therefore consider broader operating conditions and additional performance criteria, such as explicit penalties on control activity, saturation frequency and robustness to parameter variations. Moreover, other multi-objective optimizers and selection strategies can be explored and compared within the same framework. Finally, extending the approach to alternative control structures such as gain-scheduled LQR, $H_{\infty }$ control, MPC, or non-linear robust controllers and validating the tuned designs through real-time experiments on the three-DOF hover setup could be directions to further examine practicality and generalization.

## Figures and Tables

**Table 1:** System parameters.

Symbol	Description	Value	Unit
$K_{t}$	Torque thrust constant of motor/propeller	0.0036	N·m/V
$K_{f}$	Force thrust constant of motor/propeller	0.1188	N/V
$\bar{L}$	Distance between pivot to each motor	0.197	m
$m_{\mathrm{hover}}$	Total moving mass of the system	2.85	kg
$J_{y}$	Equivalent moment of inertia about yaw axis	0.110	kg·m^2^
$J_{p}$	Equivalent moment of inertia about pitch axis	0.0552	kg·m^2^
$J_{r}$	Equivalent moment of inertia about roll axis	0.0552	kg·m^2^

**Table 2:** Baseline values and search bounds for the diagonal LQR weighting parameters.

	$q_{11}$	$q_{22}$	$q_{33}$	$q_{44}$	$q_{55}$	$q_{66}$	$r_{11}$	$r_{22}$	$r_{33}$	$r_{44}$
Quanser [30]	500	350	350	0	20	20	0.01	0.01	0.01	0.01
Min	1	1	1	1	1	1	0	0	0	0
Max	1000	1000	1000	500	500	500	2	2	2	2

**Table 3:** Reproducibility details for the MOGWO optimizer.

Item	Setting
Number of search agents (wolves), $N_{w}$	80
Maximum iterations, $N_{\mathrm{it}}$	100
Archive size, $N_{A}$	100
Grid inflation parameter, α	0.1
Number of grids per objective dimension, $n_{\mathrm{Grid}}$	10
Leader selection pressure, β	4
Archive deletion pressure, γ	2

**Table 4:** Optimized LQR weighting parameters.

Method	Q (Diagonal Terms)						R (Diagonal Terms)			
	$q_{11}$	$q_{22}$	$q_{33}$	$q_{44}$	$q_{55}$	$q_{66}$	$r_{11}$	$r_{22}$	$r_{33}$	$r_{44}$
${\mathrm{MOGWO}}_{ITAE}$	464.8911	608.0898	300.0000	66.0831	10.0000	10.0000	0.0100	0.0100	0.0338	0.0122
${\mathrm{MOGWO}}_{IAE}$	414.7411	611.2435	466.9038	30.9068	10.0126	27.8179	0.0168	0.0105	0.0122	0.0160
${\mathrm{MOGWO}}_{ITSE}$	323.0642	300.0000	351.1705	43.9989	10.0000	10.0000	0.0100	0.0100	0.0299	0.0100
${\mathrm{MOGWO}}_{ISE}$	570.4597	425.9612	469.4357	11.5925	22.9484	10.4634	0.0209	0.0120	0.0428	0.0112
Quanser	500.0000	350.0000	350.0000	0.0000	20.0000	20.0000	0.1000	0.1000	0.1000	0.1000

**Table 5:** Error index values computed from the step responses.

Method	ITAE			IAE			ITSE			ISE		
	Roll	Pitch	Yaw	Roll	Pitch	Yaw	Roll	Pitch	Yaw	Roll	Pitch	Yaw
${\mathrm{MOGWO}}_{ITAE}$	0.0537	0.0300	0.2489	0.2565	0.1992	0.5890	0.0175	0.0110	0.0996	0.1512	0.1257	0.3822
${\mathrm{MOGWO}}_{IAE}$	0.0700	0.0288	0.1845	0.2758	0.1958	0.5154	0.0192	0.0107	0.0794	0.1494	0.1244	0.3412
${\mathrm{MOGWO}}_{ITSE}$	0.0455	0.0495	0.2531	0.2338	0.2474	0.6009	0.0144	0.0163	0.1056	0.1367	0.1485	0.3946
${\mathrm{MOGWO}}_{ISE}$	0.0420	0.0716	0.1812	0.2224	0.2921	0.5069	0.0129	0.0222	0.0770	0.1298	0.1718	0.3450

**Table 6:** Step-response metrics compared for all controllers.

Method	Settling Time [s]			Overshoot [%]		
	Roll	Pitch	Yaw	Roll	Pitch	Yaw
${\mathrm{MOGWO}}_{ITAE}$	0.8356	0.6007	1.6374	0.0251	0.0000	0.0000
${\mathrm{MOGWO}}_{IAE}$	1.0095	0.5772	1.2698	0.0000	0.0001	0.4197
${\mathrm{MOGWO}}_{ITSE}$	0.7788	0.8046	1.6046	0.0302	0.0000	0.0109
${\mathrm{MOGWO}}_{ISE}$	0.7527	0.9810	1.6835	0.1275	0.0007	2.5357
GA [12]	1.8363	0.7751	1.9705	0.0000	0.1105	3.7149
PSO [12]	0.4971	0.7458	1.7566	1.8152	0.0890	3.3198
SA [12]	1.5355	0.6167	1.6074	19.2070	0.0303	3.3636
GWO [12]	0.8618	0.6611	1.6270	4.1005	0.0516	3.2381
Quanser	0.9950	1.0138	1.5131	0.0000	0.0000	3.6797

**Table 7:** Control effort metrics obtained from the motor signals.

Motor	Metric [V]	${\mathrm{MOGWO}}_{\mathrm{ITAE}}$	${\mathrm{MOGWO}}_{\mathrm{IAE}}$	${\mathrm{MOGWO}}_{\mathrm{ITSE}}$	${\mathrm{MOGWO}}_{\mathrm{ISE}}$
Front Motor	Min	0.6181	1.0574	0.8798	0.8745
	Peak	2.7367	2.6072	2.2691	2.3519
	Mean	2.0106	2.0115	2.0052	2.0104
	RMS	2.0450	2.0295	2.0298	2.0395
Back Motor	Min	0.0000	0.0000	0.0000	0.0000
	Peak	2.3719	2.2869	2.3278	2.4135
	Mean	2.0106	2.0115	2.0052	2.0104
	RMS	2.0756	2.0631	2.0621	2.0753
Right Motor	Min	1.8278	1.7134	1.8369	1.7648
	Peak	3.7970	6.0424	3.8541	3.9189
	Mean	2.0133	2.0109	2.0076	2.0064
	RMS	2.0346	2.0750	2.0278	2.0330
Left Motor	Min	0.8180	1.4648	0.2415	0.1065
	Peak	2.6685	3.2128	2.7499	2.9607
	Mean	2.0133	2.0108	2.0076	2.0064
	RMS	2.0262	2.0339	2.0277	2.0359

## Data Availability

The original contributions presented in this study are included in the article. Further inquiries can be directed to the corresponding author.
